# Health-related quality of life, fear of COVID-19, and work activity among informal workers in Bogotá, Colombia

**DOI:** 10.47626/1679-4435-2024-1309

**Published:** 2025-08-25

**Authors:** Jaime Moreno-Chaparro, Olga Beatriz Guzmán-Suárez, Mónica Bermúdez-Lugo

**Affiliations:** 1 Occupation and Social Inclusion Research Group, Faculty of Medicine, Universidad Nacional de Colombia (UNAL), Bogotá, Colombia; 2 Human Occupation Department, Faculty of Medicine, UNAL, Bogotá, Colombia

**Keywords:** health status, fear, quality of life, informal sector, COVID-19

## Abstract

**Introduction:**

The COVID-19 pandemic had an impact on health and self-perception, forced us to find new forms of social interaction due to the fear of contagion, and changed our way of working and our quality of life.

**Objectives:**

To determine the relationship between health-related quality of life, fear of COVID-19, and work activities in a group of informal workers (street vendors) during the pandemic in Bogotá, Colombia.

**Methods:**

Mixed-methods study was conducted in which the European Quality of Life 5-Dimension 5-Level Scale, European Quality of Life Visual Analogue Scale, and Fear of COVID-19 Scale questionnaires, as well as a semi-structured interview, were administered in December 2021 to a group of street vendors in Bogotá. A descriptive sociodemographic analysis, a differential analysis by subgroups, and a qualitative phenomenological analysis of the underlying conditions in the quality of life-health-work activity relationship were performed.

**Results:**

Overall, 191 street vendors included. The values of the European Quality of Life 5-Dimension 5-Level Scale and European Quality of Life Visual Analogue Scale scores were higher in men (p = 0.05), and scores decreased with increasing age. The mean score on the Fear of COVID-19 Scale was 17.2, ranging from 7-34 points, suggesting a mild to moderate level of fear. Qualitative analyses revealed perceptions related to daily life, basic needs, survival, and resilience.

**Conclusions:**

The results demonstrate differences between sex, age, and length of time working on the street. At the time of the survey, fear of the virus was lower due to the exacerbation of informal workers’ needs and priorities. In this new context, they are exposed to greater vulnerability and are forced to give preference to work over health.

## INTRODUCTION

At the end of 2019, a new type of pneumonia with no obvious cause was first detected in Wuhan, China. Then, the emergence of a new infectious disease known as COVID-19, resulting from infection with a new coronavirus (SARS-CoV-2), was reported. This new disease, given its rapid spread, was soon declared a pandemic and so far has had a serious impact worldwide, both economic and in terms of public health.^1^,^2^,^3^,^4^ As of November 2022, according to official data on the COVID-19 pandemic, worldwide there had been more than 641 million COVID-19 cases, at least 6 million people had died due to COVID-19, and more than 12 billion vaccines had been administered.^5^

As time has passed, and given that both the rapid spread of the virus and the risk of developing severe COVID-19 have been controlled (both through massive vaccination and the implementation of biosecurity measures), a set of adaptive responses of mankind has emerged, including the recognition of a post-COVID-19 syndrome^6^ and naming the period after the pandemic, or at least its most critical stage, as “new normal” times.^7^ In this sense, “new normal” or “new normality” is acknowledged as a period in which normal activities are resumed within society following certain biosecurity and containment measures, together with access to vaccination, but people are still exposed to the virus, since the risk of being infected is still present. It is also understood as a period in which the concept of quality of life (QoL), the individual-society relations, and the perspectives on the future are adapted and modified.^7^,^8^

QoL in the context of the COVID-19 pandemic has been addressed in the literature based on different aspects and measurement methods, but the general consensus is that most people perceive a reduction in their QoL due to a combination of personal, environmental, and contextual factors taking place during the pandemic period.^9^,^10^,^11^,^12^ Currently, health-related QoL (HRQoL), defined as the subjective, dynamic, and multidimensional perception of people about their health and well-being,^13^ has become a central topic of study during the pandemic, as it has been traditionally considered a predictor of disease and death.^14^ Despite many studies addressing this topic have been published during the pandemic, there are still some gaps regarding the understanding of how HRQoL-related aspects vary in different populations, conditions (specific inequities, vulnerabilities, and needs), and contexts.^11^,^12^

Besides HRQoL, the pandemic has also affected people in terms of their emotional state and biopsychosocial well-being.^15^,^16^ One of the most common emotions during the pandemic was fear, defined as feeling in danger by an external stimulus, which in the case of the COVID-19 crisis led to several physiological, cognitive, physical, and social responses.^16^,^17^ Likewise, it should be noted that these concepts and their relationship among each other are potential modifiers of several aspects, including health state, well-being, and social participation, among others.

Thus, as in the case of HRQoL, the current situation implies not only understanding fear in the “new normal” times, but also approaching the new dynamics of everyday life taking into account that both in-person education and work activities must continue, that some population groups are at a higher risk of developing severe complications in case of getting infected with the virus, and that, depending on the regulations, restrictions, and needs of each country, the phenomenon to be addressed may vary.

One of such population groups is informal workers, specifically street vendors, who were highly affected in the context of the pandemic, as their work activity was seriously affected by the human mobility restrictions imposed by most countries to control the spread of the virus, including social distancing measures, compliance with strict and costly biosecurity standards, and the inability to sell their goods in public spaces as they usually did.^18^ Informal work is defined as any work activity that is not regulated by labor law or a competent official authority and in which a person, with or without any payment, provides a good or a service. Therefore, informal workers are not entitled to fair working conditions, are not entitled to be enrolled in health care or other social protection systems by their employers, and are defenseless against work-related risks or changes, such of those resulting from health crisis such as a pandemic.^19^ In Colombia, informal workers account for 46.8% of all workers in the country; besides, by 2021, informal work was the main means of earning money in face of the shortage of both jobs and money caused by the COVID-19 pandemic.^20^

Taking this into account, the aim of this study was to understand the relationship between HRQoL, fear of COVID-19, and work activities in a group of informal workers (street vendors) amidst the pandemic in Bogotá, Colombia.

## METHODS

### ETHICAL CONSIDERATIONS

The study was approved by the Ethics Committee of the Faculty of Medicine of the Universidad Nacional de Colombia (UNAL), as stated in Minutes 009-067, issued on May 13, 2021. All participants signed the respective informed consent form and data anonymization, ensuring confidentiality at all times.

### DESIGN AND STUDY PARTICIPANTS

A mixed-methods cross-sectional study was conducted, in which informal workers selling goods in public spaces of Bogotá D.C. (street vendors) were recruited between November and December 2021, using a non-probabilistic sampling method based on geographic density and participation quotas. The research team identified the geographic areas and specific locations within Bogotá with the highest densities of informal workers.

Participants were selected according to the following criteria: 1) the probability of finding one or more street vendors in one square meter (m^2^) of public space; 2) a non-response rate of 5%; and 3) the fact that only street vendors selling food, miscellaneous items (electronic items, hardware items, stationery items, others), or clothes were considered. Likewise, the following inclusion criteria were considered: 1) having worked for at least 5 years as a street vendor selling goods informed in the identification process (seniority); 2) having worked for at least 5 years in the location where the informal worker was identified; 3) being older than 18 years; 4) being officially registered in the Institute for Social Economy of Bogotá (*Instituto para la Economia Social* [IPES]) as a street vendor through the completion of the Informal Street Vendor Individual Registration Form; and 5) having a score < 20 points in the System for the Identification of Potential Beneficiaries of Social Protection Programs (*Sistema de Identificación de Potenciales Beneficiarios de Programas Sociales* [SISBÉN]), a Colombian system developed by the *Departamento Administrativo Nacional de Estadística* (DANE) to measure the socioeconomic vulnerability of people.

### MEASUREMENTS

A culturally and linguistically validated version of the European Quality of Life 5-Dimension 5-Level (EQ-5D-5L) Scale questionnaire (license no. 45999), created by the EuroQoL Group^21^ for the Colombian population (mean general index 0.953), was used to collect data on HRQoL.^21^,^22^ This questionnaire addresses five domains (mobility, self-care, activities of daily living, pain/ discomfort, and anxiety/depression), and respondents are asked to rate each domain using a 1-5 scale; thus, 3,125 possible health states, ranging from no problems in any dimension (11111) to extreme problems in all domains (55555), can be established.^22^ Additionally, the European Quality of Life Visual Analogue Scale (EQVAS) was administered in order to obtain a subjective rating (0 to 100) on the health status of each respondent.^21^,^22^

On the other hand, the Spanish version of the Fear of COVID-19 Scale (FCV-19S) was also used. This instrument was initially developed in Iran,^23^ but it has been translated and validated in different Spanish-speaking countries such as Peru,^24^ Argentina,^25^ Ecuador,^26^ and Colombia^27^ with good psychometric results (Cronbach’s alpha value of 0.75 and MacDonald’s omega of 0.78). The FCV-19S allows assessment of thoughts, emotions, behaviors, and physiological responses in relation to COVID-19 using a 5-point Likert scale (1 = strongly disagree, 5 = strongly agree), and a score > 7 is interpreted as presence of fear (maximum score = 35).

Participants were also administered the Occupational History Questionnaire (OHQ), which was developed by the Department of Human Occupation of the Faculty of Medicine of the UNAL. This instrument was designed to be used in the context of a qualitative interview based on a phenomenological-interpretative perspective that aims to collect information to build concepts and occupational narratives that make work-related situations and realities visible by means of the recognition, analysis, and understanding of the population and its context.

### VARIABLES

#### Sociodemographic variables

The following sociodemographic data were obtained: sex, age, educational level, place of origin, current type of residence (urban or rural), socioeconomic level (it should be noted that in Colombia there are six socioeconomic strata, which are used to determine the level of income of people as well as their access to public services, being strata 1 and 2 equivalent to low socioeconomic level, that is, the most vulnerable individuals, or those with the least access to such services, and strata 5 and 6 equivalent to high socioeconomic level), whether the worker was a victim of forced displacement, number of years working as an informal street vendor, and type of goods sold (food, miscellaneous items, and clothes).

#### European Quality of Life 5-Dimension 5-Level Scale and European Quality of Life Visual Analogue Scale

The following variables were included for analysis: those related to the five domains of the EQ-5D-5L scale (mobility, self-care, activities of daily living, pain/ discomfort, and anxiety/depression), health states with the corresponding variations, and the subjective rating of health status (EQ-VAS).

#### Fear of COVID-19 Scale

Responses about the scoring of the following dimensions were included for analysis (both as categorical and quantitative variables): thoughts, emotions, behaviors, and physiological responses in relation to COVID-19.

#### Work activity

This aspect was explored by a qualitative interview based on the OHQ, where questions were made about the impact of the COVID-19 pandemic on health status and about what participants believe the health-work activity relationship and daily life would be in the future. The following questions were made: 1) Do you think your health status changed during the pandemic? If so, in what ways did it change?; 2) Was there any issue in the health status-work activity relationship amidst the pandemic?; 3) Do you think your quality of life increased, decreased, or did not experience any change at all after the pandemic or in the context of the new normal reality?

### STATISTICAL METHODS AND ANALYSIS

Sociodemographic, EQ-5D-5L/EQ-VAS, and FCV-19S data were analyzed descriptively. Categorical and quantitative variables are expressed as absolute frequencies and percentages, and measures of central tendency and of dispersion, respectively. Analyses were performed depending on the type of goods sold by the street vendors. Mean values obtained in the questionnaires were compared to demographic variables using a t-test (two variables) or an analysis of variance (ANOVA) (three or more variables); all statistical analyses were performed using R software (version 3.5.0).^28^ Qualitative analyses of the responses obtained after the administration of the OHQ were carried out using the NVivo 12 software.^29^

## RESULTS

A total of 191 street vendors were included in the study. Participants’ mean age was 44.5 years (standard deviation [SD] = 13.6), 52.4% were women, and 40.3% were from Bogotá D.C. About their schooling level, 85.2% reported having completed any level of education; however, 14.6% reported not having any schooling at all. Furthermore, it is worth noting that 78.5 and 21.5% of the participants had a low and a middle socioeconomic status, respectively; besides, 10.5% were victims of forced displacement due to violence and internal conflict.

Regarding occupational variables, the average number of years working in the informal sector was 16.5, and there was a similar distribution of workers in the three street vending categories that were considered: food (n = 67), miscellaneous items (n = 57), and clothing (n = 67) (Table 1).

**Table 1:** Sociodemographic, socioeconomic, and occupational characteristics of the sample according to EQ-5D-5L index value and EQ-VAS scores

Sociodemographic variable	Total (n=191) n (%)	EQ-5D-5L		EQ-VAS	
		Mean (SD)	p-value	Mean (SD)	p-value
Sex			0.516		0.05
Female	100 (52.4)	0.870 (0.159)		76.6 (19.7)	
Male	91 (47.6)	0.885 (0.146)		82.0 (18.7)	
Age (years)			0.164		0.655
Mean (SD)	44.5 (13.6)				
18-24	15 (7.8)	0.944 (0.058)		86.5 (14.8)	
25-34	38 (19.9)	0.910 (0.085)		81.5 (17.4)	
35-44	44 (23.0)	0.880 (0.158)		79.0 (18.1)	
45-54	46 (24.0)	0.833 (0.186)		76.8 (23.7)	
55-64	34 (17.8)	0.831 (0.135)		76.3 (19.3)	
65-74	12 (6.28)	0.830 (0.244)		75.0 (19.1)	
> 75	2 (1.04)	0.825 (0.025)		70.0 (0.0)	
Birth department			0.166		0.04
Bogotá	77 (40.3)	0.894 (0.140)		83.2 (16.4)	
Cundinamarca	32 (16.8)	0.906 (0.122)		74.3 (20.2)	
Boyacá	10 (5.2)	0.772 (0.237)		65.0 (24.6)	
Caldas	10 (5.2)	0.870 (0.152)		66.6 (27.0)	
Valle del Cauca	8 (4.2)	0.875 (0.187)		66.1 (17.9)	
Education level			0.04		0.116
No education	28 (14.6)	0.871 (0.168)		64.0 (35.1)	
Complete elementary school	68 (35.6)	0.829 (0.195)		77.7 (19.6)	
Complete secondary or high school (baccalaureate)	71 (37.1)	0.913 (0.093)		84.0 (15.8)	
Complete technician	8 (4.2)	0.942 (0.071)		82.5 (20.5)	
Complete technologist	12 (6.2)	0.970 (0.162)		80.0 (18.3)	
Higher education	4 (2.1)	0.918 (0.054)		82.5 (12.6)	
Residence			0.412		0.729
Urban	187 (97.9)	0.926 (0.149)		79.1 (19.4)	
Rural	4 (2.1)	0.876 (0.153)		82.5 (23.6)	
Socioeconomic stratum			0.395		0.001
One	53 (27.7)	0.864 (0.174)		71.2 (22.9)	
Two	97 (50.8)	0.893 (0.158)		82.9 (17.4)	
Three	41 (21.5)	0.905 (0.103)		82.3 (16.2)	
Displacement			0.004		0.02
Yes	20 (10.5)	0.786 (0.174)		70.2 (20.4)	
No	171 (89.5)	0.888 (0.147)		80.2 (19.1)	
Years in the informal sector			0.239		0.343
Mean (SD)	16.5 (11.3)				
< 10	103 (53.9)	0.899 (0.113)		88.8 (19.2)	
11-20	35 (18.3)	0.860 (0.210)		83.7 (18.7)	
21-30	27 (14.1)	0.854 (0.145)		77.8 (18.2)	
31-40	18 (9.4)	0.854 (0.154)		72.7 (23.1)	
41-50	8 (4.2)	0.800 (0.280)		73.5 (19.0)	
Type of good sold			0.425		0.931
Food	67 (35.1)	0.857 (0.187)		79.9 (19.4)	
Miscellaneous items	57 (29.8)	0.890 (0.121)		79.0 (19.1)	
Clothing	67 (35.1)	0.885 (0.137)		78.6 (19.8)	

EQ-5D-5L = European Quality of Life 5-Dimension 5-Level Scale; EQ-VAS = European Quality of Life Visual Analogue Scale; SD = standard deviation.

It was found that men had higher EQ-5D-5L index values and EQ-VAS scores than women, being the difference significant in the latter (p = 0.05). Scores in both questionnaires decreased consistently as age increased; likewise, scores, especially in the EQ-VAS questionnaire, were lower in those who had been born in cities or regions that were not close to Bogotá, the capital city of Colombia (e.g., Boyacá, Caldas, and Valle del Cauca) (p = 0.04). Scores in both scales were lower in those vendors with a low socioeconomic stratum, which are at a higher level of vulnerability (stratum 1 vs. stratum 3), being this difference statistically significant in the case of the EQ-VAS (p = 0.001). Scores were also significantly lower in both scales (EQ-5D-5L and EQ-VAS) in informal workers who were victims of forced displacement due to violence and internal conflict (p = 0.004 and p = 0.02, respectively). In relation to their schooling level, those with a higher level of education obtained higher scores, with this difference being significant in the case of the EQ-5D-5L scale (p = 0.04); finally, no significant differences were observed in terms of the scores obtained in both scales among the three categories of street vendors that were established according to the type of goods they sold.

In relation to the health states resulting from the administration of the EQ-5D-5L questionnaire, 11111 (optimal health state) was the most common health state (36.6%). A noteworthy finding is that 6.8 and 7.3% of participants had mild to moderate anxiety/ depression, respectively, and that 2.6 and 6.8% had not only mild to moderate problems related to anxiety/ depression, but also mild to moderate pain/discomfort, respectively (Table 2).

**Table 2:** Most frequently reported European Quality of Life 5-Dimension 5-Level (EQ-5D-5L) Scale health states

EQ-5D-5L health state	n (%)
11111	70 (36.6)
11112	14 (7.3)
11113	13 (6.8)
11122	13 (6.8)
11121	11 (5.8)
11132	6 (3.1)
11131	5 (2.6)
11123	4 (2.1)
12211	4 (2.1)
Others	51 (26.7)

**Figure 1:**
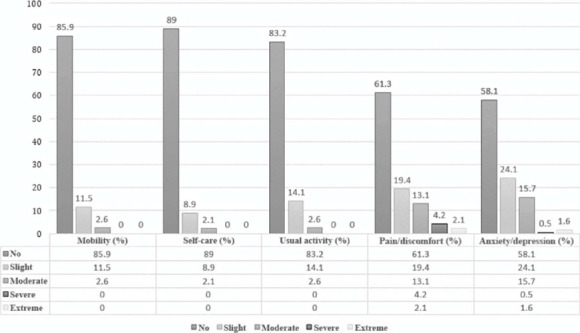
Symptomatology distribution by domain according to the European Quality of Life 5-Dimension 5-Level (EQ-5D-5L) Scale responses.

In a more detailed analysis of EQ-5D-5L health states, the following findings were observed: the highest proportion of patients reporting any symptoms was found in the anxiety/depression (41.9%) and pain/ discomfort (39.7%) domains. In addition, 24.1 and 15.7% of respondents reported they had mild and moderate symptoms, respectively, in the anxiety/ depression domain, while in the pain/discomfort domain, mild and moderate symptoms were reported by 19.4 and 13.1%, respectively. Finally, it is worth noting that 14.1 and 11.5% of the respondents reported mild symptoms in the activities of daily living and mobility or displacement domains, respectively. Health state distribution is shown in Figure 1.

On the other hand, the average score in the FCV-19S was 17.2, with an interesting range of 7 to 34 points, that is, a mild to moderate level of fear of COVID-19. According to the data shown in Table 3, respondents’ opinions were divided regarding the question about being afraid of COVID-19 (34% disagreed and 30.4% agreed). In the case of questions about feeling uncomfortable thinking of the virus, being afraid of dying because of COVID-19, becoming nervous when watching news about the virus, having sleep problems because of COVID-19 concerns, and feeling the heart race or palpitate when thinking about the disease, more than 50% of participants disagreed or strongly disagreed. However, it is worth noting that, although they were not the majority, 30.4, 29.8, 27.2, and 24.1% of participants agreed they were afraid of COVID-19, felt uncomfortable when thinking about the virus, were afraid of dying because of it, and became nervous or anxious when watching news about the disease, respectively, which shows a mental and social health trend.

**Table 3:** Overall results in each question of the FCV-19S

FCV-19S					
Mean (SD)	17.2 (6.66)				
Median (min, max)	16.0 (7, 34)				
Item	Strongly disagree (1) n (%)	Disagree (2) n (%)	Neither agree nor disagree (3) n (%)	Agree (4) n (%)	Strongly agree (5) n (%)
I1. I am most afraid of coronavirus-19.	27 (14.1)	65 (34.0)	20 (10.5)	58 (30.4)	21 (11.0)
I2. It makes me uncomfortable to think about coronavirus-19.	36 (18.8)	68 (35.6)	16 (8.4)	57 (29.8)	14 (7.3)
I3. My hands become clammy when I think about coronavirus-19.	54 (28.3)	101 (52.9)	10 (5.2)	23 (12.0)	3 (1.6)
I4. I am afraid of losing my life because of coronavirus-19.	38 (19.9)	63 (33.0)	14 (7.3)	52 (27.2)	24 (12.6)
I5. When watching news and stories about coronavirus-19 on social media, I become nervous or anxious.	37 (19.4)	79 (41.4)	16 (8.4)	46 (24.1)	13 (6.8)
I6. I cannot sleep because I’m worrying about getting coronavirus-19.	56 (29.3)	101 (52.9)	13 (6.8)	19 (9.9)	2 (1.0)
I7. My heart races or palpitates when I think about coronavirus-19	53 (27.7)	98 (51.3)	11 (5.8)	23 (12.0)	6 (3.1)

FCV-19S = Fear of COVID-19 Scale; SD = standard deviation.

In the case of the FCV-19S questionnaire, in five out of seven questions, women had a higher mean response rate and, therefore, a higher perception of fear of COVID-19. In terms of age groups, the highest scores were found in those aged 35-44 (3.16) and 65-74 (3.17) years in the question about the general perception of fear of COVID-19, while scores were similar in all age groups in the question related to fear of dying because of being infected with the virus (2.67; 3.27). Similar to what was observed in the case of the scores in the EQ-5D-5L and the EQ-VAS, scores were higher in all questions of the FCV-19S in participants with a higher level of education; a trend that was also observed in those with a low socioeconomic status (and, consequently, a higher vulnerability), those who were victims of forced displacement (with significant differences in questions I1 [p = 0.05] and I2 [p = 0.04]), and those with a higher number of years working in the informal sector (with significant differences in questions I2 and I3 [p = 0.04]) (Table 4).

**Table 4:** Distribution of sociodemographic variables according to FCV-19S scores

Sociodemographic variables	FCV-19S						
	I1	I2	I3	I4	I5	I6	I7
Sex							
Female	2.96 (1.24)	2.73 (1.22)	2.04 (1.00)	2.93 (1.37)	2.61 (1.23)	1.97 (0.90)	2.20 (1.14)
Male	2.84 (1.34)	2.69 (1.34)	2.08 (0.95)	2.65 (1.36)	2.54 (1.25)	2.04 (0.95)	2.01 (0.93)
p-value	0.504	0.839	0.796	0.156	0.691	0.583	0.214
Age (years)							
18-24	2.73 (1.16)	2.87 (1.30)	1.73 (0.79)	3.27 (1.22)	2.33 (1.05)	1.87 (0.74)	2.13 (0.74)
25-34	2.61 (1.20)	2.71 (1.39)	1.95 (0.98)	2.71 (1.41)	2.37 (1.20)	1.97 (0.85)	2.11 (1.03)
35-44	3.16 (1.36)	2.80 (1.21)	2.20 (1.00)	2.86 (1.29)	2.68 (1.22)	2.09 (0.85)	2.20 (1.00)
45-54	2.85 (1.32)	2.63 (1.31)	2.07 (1.08)	2.80 (1.46)	2.67 (1.35)	2.02 (1.04)	2.17 (1.25)
55-64	2.94 (1.35)	2.76 (1.30)	2.07 (1.08)	2.76 (1.39)	2.71 (1.29)	2.12 (1.12)	2.15 (1.08)
65-74	3.17 (1.11)	2.58 (1.08)	1.92 (0.79)	2.67 (1.44)	2.50 (1.24)	1.58 (0.51)	1.50 (0.52)
> 75	3 (1.41)	1.50 (0.70)	1.50 (0.70)	3.00 (1.41)	2.00 (0.64)	2.00 (0.00)	1.50 (0.70)
p-value	0.590	0.863	0.576	0.878	0.800	0.715	0.508
Education level							
No education	2.80 (1.10)	2.80 (1.10)	2.80 (1.10)	2.80 (1.10)	3.00 (1.41)	2.40 (0.89)	
Complete elementary school	3.08 (1.28)	3.04 (1.33)	2.21 (0.97)	2.83 (1.34)	2.79 (1.25)	2.38 (1.31)	2.33 (1.24)
Secondary or high school	2.57 (1.27)	2.49 (1.28)	1.87 (0.87)	2.75 (1.39)	2.35 (1.16)	1.83 (0.75)	1.96 (0.89)
Complete technician	3.25 (1.49)	2.50 (1.60)	2.00 (1.31)	3.00 (1.93)	3.25 (1.67)	2.38 (1.06)	2.63 (1.30)
Complete technologist	3.14 (1.21)	3.43 (1.13)	2.00 (1.15)	2.96 (1.57)	2.29 (1.38)	1.86 (1.21)	2.14 (1.46)
Complete professional	3.25 (0.50)	2.25 (1.26)	1.75 (0.50)	2.00 (0.83)	2.00 (0.83)	2.00 (0.00)	1.75 (0.50)
p-value	0.178	0.421	0.116	0.985	0 .374	0.147	0.624
Socioeconomic stratum							
One	3.04 (1.19)	2.79 (1.29)	2.21 (0.96)	3.04 (1.39)	2.79 (1.23)	2.19 (0.98)	2.43 (1.17)
Two	2.92 (1.40)	2.68 (1.25)	1.93 (1.08)	2.65 (1.29)	2.45 (1.22)	1.93 (0.84)	1.98 (0.98)
Three	2.68 (1.11)	2.68 (1.33)	2.03 (0.94)	2.83 (1.50)	2.59 (1.26)	1.95 (1.02)	2.00 (1.00)
p-value	0.409	0.866	0.362	0.248	0.277	0.236	0.020
Displacement							
Yes	3.40 (1.23)	3.25 (1.21)	2.40 (0.99)	3.20 (1.36)	2.75 (1.29)	2.20 (0.95)	2.40 (1.10)
No	2.84 (1.28)	2.65 (1.27)	2.02 (0.97)	2.75 (1.36)	2.56 (1.23)	1.98 (0.92)	2.08 (1.04)
p-value	0.050	0.040	0.090	0.163	0.5 07	0.322	0.192
Years in the informal sector							
< 10	2.83 (1.22)	2.62 (1.24)	1.93 (0.88)	2.83 (1.36)	2.52 (1.18)	1.92 (0.82)	1.99 (0.94)
11-20	3.14 (1.42)	2.91 (1.34)	2.40 (1.22)	2.89 (1.45)	2.83 (1.36)	2.17 (1.10)	2.29 (1.18)
21-30	3.04 (1.40)	2.90 (1.27)	2.26 (1.02)	2.59 (1.19)	2.59 (1.37)	2.04 (0.94)	2.33 (1.11)
31-40	2.33 (1.19)	3.33 (1.41)	2.72 (0.75)	2.33 (1.46)	2.28 (1.18)	2.11 (1.13)	2.22 (1.35)
41-50	3.63 (0.74)	3.88 (0.35)	3.25 (0.88)	3.63 (1.30)	2.75 (1.16)	2.00 (0.92)	1.88 (0.64)
p-value	0.091	0.040	0.040	0.210	0.5 87	0.700	0.387

FCV-19S = Fear of COVID-19 Scale.

To conclude the approach to the current phenomenon, the qualitative analysis of the work activity of the participants was conducted through first-level coding and phenomenological interpretations of the questions made in the interviews. With this purpose in mind, literal transcriptions exceeding 40,000 words of the semi-structured interviews were made, and data saturation was reached in participant number 23 (of 191). The following categories of analysis were defined: 1) Changes in health status due to the pandemic; 2) Changes in health status due to work or vice versa; and 3) QoL amidst the pandemic and perception about the future.

### Changes in health status due to the pandemic

Undoubtedly, the main negative effect of the pandemic on health is the virus itself; however, for the purposes of the present study, we asked participants about other health effects that were related to the daily life of street vendors in the context of the pandemic and the new normality. In this sense, other health-related aspects were identified, such as the following relationships: obesity-sedentary lifestyle-diet, anxiety-depression-mental health, and sleep-wakefulness, among others.

P20 *“Well, of course we [street vendors] experienced changes, we gained weight because we could not go out; we could not stop thinking about what we were going to do to earn a living the next day, since everything was closed; we felt fear and anxiety about the next day and the next month [...] in the end we felt uneasy because we did not know how to get the money for our basic needs every day, we were not able to sleep well due to such concerns, we had to eat whatever we could afford as we could not sell any goods in the streets and make a living, and above all we were worried about not getting infected with virus and end up hospitalized”* (43-year old woman, street vendor selling miscellaneous items, Santa Fe district).

### Changes in health due to work or vice versa

Regular work activity implies a series of exposures to occupational risks that determine, in the short or the long term, the probability of suffering an occupational accident or disease. In this sense, we aimed at inquiring about the relationship between work and health in the context of the pandemic, that is, whether one prevailed over the other or if they were in contradiction. It became evident that, at the time interviews were conducted, working to earn the money necessary to afford daily basic needs was more important than health status or even the risk of exposure to the virus.

P3 *“We [street vendors] had to decide whether we had to work and earn some money or to get sick, because given our current situation, we have to think first in our daily needs, in bringing money to the household, then we can think about our own health. The saddest and most frightening situation was what we had to do with the elderly, we had to lock them at home and keep them away so that they would not get sick. [...] In case we [street vendors] got infected with the virus, then we had to go home and as soon as we had recovered, we returned to the streets”* (30-year-old man, informal worker selling clothes, Kennedy district).

### Quality of life amidst the pandemic and perception about the future

Questions about QoL were made to establish a qualitative comparison between the beginning of the pandemic and the new normality and considering the priorities of street vendors in relation to this concept and their reality. A sustained tendency towards prioritizing food, housing, health, the need for survival, and resilience was shown.

P15 *“I understand quality of life as having a meal, a place to live, being in good health and with God. During the pandemic we were hungry a lot of times, we were afraid, we did what we could, I was one of those people who put red flags made with rags to receive aid from the State. Now that we are overcoming the virus, we must work, take care of ourselves, and recover what we lost”* (55-year-old woman, street vendor selling food, San Cristóbal district).

## DISCUSSION

The present study provides information that contributes to the understanding of the relationship between HRQoL, fear of COVID-19, and work activities in the context of the so-called new normality due to the COVID-19 pandemic in street vendors, a highly vulnerable and affected population group in a middle-income country like Colombia.

As for QoL and HRQoL, scores in the EQ-5D-5L and EQ-VAS questionnaires were higher in men. Besides, a decreasing trend was observed as age increased; likewise, scores were lower in street vendors who were born in areas that were not close to the central region of the country, in those with a low socioeconomic status (i.e., with a higher level of vulnerability) and in those who were victims of forced displacement due to violence. On the contrary, higher scores, especially in the case of the EQ-5D-5L questionnaire, were observed in participants with a higher level of education. Our data can be compared with those reported by Bailey et al., who, in a study that addressed the population norms and health inequality in Colombia based on the EQ-5D-5L instrument, found that men had better scores and higher maximum levels than women (EQ-VAS score of 86.3 vs. 83.9/index value of 0.957 vs. 0.949) and that these scores showed a decreasing trend as age increased (EQ-VAS score of 89.1 [18 to 24 years] vs. 80.8 [55 to 64 years]/index value of 0.970 [18 to 24 years] vs. 0.927 [55 to 64 years]) and income decreased (EQ-VAS value of 88.0 [higher socioeconomic stratum] vs. 83.9 [lower socioeconomic stratum]/ index 0.960 [higher socioeconomic stratum] vs. 0.948 [lower socioeconomic stratum]).^22^ Compared to our study, significant differences were not found between technicians and university graduates, but scores consistently increased as the level of schooling increased. Finally, these data confirm that these scores have a lower tendency in Colombia compared to other countries. In this regard, conducting broader surveys, including people from all socioeconomic strata in Colombia, would surely be useful to confirm this information.

In the case of health states, we found that optimal health (state 11111) was the most frequent among participants (36.6%); however, it should be noted that some of the participants reported having some mild to moderate symptoms in the anxiety/depression (7%) and pain/discomfort (2%) domains. Our results are in line with those reported in the study by Bailey et al.,^22^ where optimal health state was also the most frequent, and health problems were mostly found in the anxiety/depression (9%) and pain/discomfort (4%) domains.

The mean score in the fear of COVID-19 category, as evaluated with the FCV-19S, was 17.2, with ranging scores suggestive of mild to moderate fear of COVID-19, even in new normal times. Based on these results, it was observed that despite most social distancing and biosecurity restrictions have been lifted, fear of COVID-19 is still present. Likewise, although more than half of the participants reported they were not afraid of the virus or felt uncomfortable thinking about it, the persistence of responses involving altered states of mental and social health was evidenced. The latter finding could be associated with the results observed in the administration of the EQ-5D-5L questionnaire, where the presence of anxiety/depression stood out. Finally, higher scores of fear were found among: 1) street vendors older than 35 years, but especially those over 65 years; 2) those with higher schooling levels; 3) those highly vulnerable; and 4) those who were victims of forced displacement due to violence.

The above results can be compared with those reported by Sotomayor-Beltran et al.^30^ in a study in which the same questionnaire was administered to people living in disadvantaged communities of Peru. Although in the study by Sotomayor-Beltran et al.^30^ the sample was not specifically composed of street vendors, similar sociodemographic data were evidenced, such as predominance of women (51.2%), average age over 30-40 years, educational disadvantages, and level of income. As for the FCV-19S, the mean score in the study conducted in Peru (24.0 ± 4.94) was higher than the one observed in our study (17.2), and they also found that the abovementioned sociodemographic variables did determine changes in said score. Finally, the most important finding in the study conducted by Sotomayor-Beltran et al.^30^ was that those who were formal workers and those who had completed workforce education were more likely to be afraid of COVID-19 (odds ratio [OR]: 13.22 [95%CI 4.00-43.62] and OR: 27.07 [95%CI 1.61-454.24], respectively) compared to other workers (such as informal workers, in our case). The fact that the study conducted in Peru included distinct types of workers, although with similar characteristics to our sample in terms of vulnerability, might explain why this score was lower in our study.

Other comparisons, in this case indirect (due to the use of other scales but with the same measurement intention) are those related to the following studies: first, the study conducted by Romero-Michel et al.^31^ in Mexico, in which a group of street vendors were found to have low levels of concern about SARS-CoV-2 (they were 11 times more likely to have no concern or fear of COVID-19 compared to formal workers), but a latent anxiety about their health and economic situation was evident in said group. In the same line, a similar study found that, compared to formal workers, street vendors would rather be exposed to the virus than being unable to work at all (OR: 19.4, [95%CI 4.6-81.7]; p < 0.001) and that their lack of concern about the increasing number of COVID-19 cases was greater than the one observed in formal employees (OR: 0.06 [95%CI 0.01-0.3]; p = 0.002).^32^

Second, a study carried out by Martínez et al.^33^ in Cali, Colombia, where, by means of telephone-based surveys, community leaders and street vendors were asked, among other things, about their sociodemographic characteristics, found that women were predominant (49%), most participants were socioeconomically vulnerable (80%), a high proportion (97%) was enrolled in the subsidized health care system (63%), and almost all (97%) reported having experienced a reduction in their income due to the pandemic. In addition, said study found that the average life satisfaction score was 6.9 on a 0-10 scale, that anxiety and stress levels were high (average score of 6.8 on a 0-10 scale), and that the level of general concern about well-being was also elevated (mean score of 6.3). Martínez et al.^34^ also observed that the most frequently reported consequences of the pandemic on economic activity (with an emphasis put on fear) were the risk of death of any family member (66%), getting infected with the virus and getting sick (43%), and experience economic crisis at home (51%). Likewise, subjective well-being was identified as ranging between good and very good (75%), and the mean score in the anxiety and stress domain was 6.5 on a 0-10 scale.

Third, the research carried out by Haque et al.,^35^ by means of the administration of the General Health Questionnaire (GHQ-12) to informal garbage workers from Bangladesh, found that 80.6% of them suffered from psychological distress (overall psychological stress score: 15.6 ± 3.4) and that 67.6% reported symptoms of anxiety/depression. These results were in turn related to informal workers with low levels of schooling, of monthly income, and living in disadvantaged communities, among other factors.

Fourth, the study by Swarna et al.^36^ administered a questionnaire to 1,867 informal workers in Bangladesh and found significant results. These included a reduced income (98%) and decreased food expenditure (95%), along with a negative perception of health and well-being, which was associated with poverty, lower daily income, and sadness.

The results of the qualitative approach of the present study reveal an even bigger picture. On the one hand, other health problems resulting from the pandemic, such as the obesity-sedentary lifestyle-diet, anxiety-depression-mental health, and sleep-wakefulness relationships (all of which have repercussions not only on mental but also on physical health and social well-being), are identified; on the other, changes in the work dynamics of these workers that were mediated by necessity, prioritization of food and household maintenance, social survival and the now obligatory nature of resilience, and adjustment to a new way of life. The above data can be compared with those reported by Singh & Kaur^37^ in a study conducted in Punjab, India, where narratives of female informal workers were collected, evidencing different factors affecting them in terms of health, working conditions, and everyday life in general, including poverty, food insecurity, inflexible restrictions and rules implemented to control the spread of the pandemic, and social prejudices about informal work activities and being a woman.

Regarding the limitations of this study, it is worth noting that it was conducted in a period when social distancing and biosecurity measures due to the pandemic had been lifted in the context of the new normality. Moreover, only street vendors working in Bogotá were included; thus, these results might not be generalized for street vendors in other cities and regions of the country. Furthermore, because of the study design, causal association possibilities are limited; however, the findings reported here could be used to conduct a wide range of possible novel studies. Finally, as the data analyzed here were self-reported, there is a possibility of information or memory bias. Regarding its strengths, it should be noted that this study integrates a quantitative and qualitative vision of a phenomenon that has been scarcely addressed, it provides information of a vulnerable and highly segregated population group in society, and, finally, it constitutes a significant advance towards considering modifications and transformations in terms of the health, well-being, and work activity of street vendors.

## CONCLUSIONS

This study revealed a series of relationships between sociodemographic variables, the perception and state of health/well-being, fear of COVID-19, and work activity amidst the pandemic and the so-called new normal times in street vendors. This new period implies several problems for these workers, including increased vulnerability, the need to prioritize their daily subsistence even by putting their health and well-being aside, and the preponderance of a set of factors that perpetuate the conditions of inequality and social inequity to which they are exposed, namely: adverse economic situation, poor working conditions, poor access to mental health services, and the impossibility of accessing job opportunities in line with such new normality.
